# Plasminogen activator urokinase interacts with the fusion protein and antagonizes the growth of Peste des petits ruminants virus

**DOI:** 10.1128/jvi.00146-24

**Published:** 2024-03-05

**Authors:** Junhuang Wu, Wenping Yang, Lingxia Li, Jingyan Wu, Jijun He, Yi Ru, Jingjing Ren, Yong Wang, Haixue Zheng, Youjun Shang, Dan Li

**Affiliations:** 1 State Key Laboratory for Animal Disease Control and Prevention, College of Veterinary Medicine, Lanzhou University, Lanzhou Veterinary Research Institute, Chinese Academy of Agricultural Sciences, Lanzhou, China; 2 Gansu Province Research Center for Basic Disciplines of Pathogen Biology, Lanzhou, China; 3 College of Agriculture and Animal Husbandry, Qinghai University, Xining, China; 4 College of Animal Science and Technology, Anhui Agricultural University, Hefei, China; Loyola University Chicago - Health Sciences Campus, Maywood, Illinois, USA

**Keywords:** Peste des petits ruminants virus, fusion protein, plasminogen activator urokinase, VISA

## Abstract

Peste des petits ruminants is an acute and highly contagious disease caused by the Peste des petits ruminants virus (PPRV). Host proteins play a crucial role in viral replication. However, the effect of fusion (F) protein-interacting partners on PPRV infection is poorly understood. In this study, we found that the expression of goat plasminogen activator urokinase (PLAU) gradually decreased in a time- and dose-dependent manner in PPRV-infected goat alveolar macrophages (GAMs). Goat PLAU was subsequently identified using co-immunoprecipitation and confocal microscopy as an F protein binding partner. The overexpression of goat PLAU inhibited PPRV growth and replication, whereas silencing goat PLAU promoted viral growth and replication. Additionally, we confirmed that goat PLAU interacted with a virus-induced signaling adapter (VISA) to antagonize F-mediated VISA degradation, increasing the production of type I interferon. We also found that goat PLAU reduced the inhibition of PPRV replication in VISA-knockdown GAMs. Our results show that the host protein PLAU inhibits the growth and replication of PPRV by VISA-triggering RIG-I-like receptors and provides insight into the host protein that antagonizes PPRV immunosuppression.

**IMPORTANCE:**

The role of host proteins that interact with Peste des petits ruminants virus (PPRV) fusion (F) protein in PPRV replication is poorly understood. This study confirmed that goat plasminogen activator urokinase (PLAU) interacts with the PPRV F protein. We further discovered that goat PLAU inhibited PPRV replication by enhancing virus-induced signaling adapter (VISA) expression and reducing the ability of the F protein to degrade VISA. These findings offer insights into host resistance to viral invasion and suggest new strategies and directions for developing PPR vaccines.

## INTRODUCTION

Peste des petits ruminants virus (PPRV) is a single-stranded, negative-sense RNA virus that is highly pathogenic and causes a contagious disease in sheep and goats (1). It is classified as a category A highly contagious animal disease that often presents with symptoms such as high fever, oral ulcers, and inflammation (2, 3). For strains with high pathogenicity, the mortality rates can reach 100%. PPRV continues to have a global impact, affecting captive and wild animals and resulting in significant biodiversity loss and economic damage worldwide(4).

The PPRV genome is approximately 15,948 nucleotides long and encodes eight proteins. These include six structural proteins: nucleocapsid (N), phosphoprotein (P), matrix (M), fusion (F), hemagglutinin (H), and polymerase (L), and two non-structural proteins, C and V (5). Certain viral proteins, such as the V protein, inhibit the host’s innate immune response by blocking the nuclear translocation of STAT1/2 proteins (6). N protein, a conserved structural protein with high immunogenicity, can also disrupt the formation of TANK-binding kinase 1 (TBK1) and interferon regulatory factor 3 (IRF3) complexes, thereby inhibiting the production of type I interferon (IFN-I) (7). N and P proteins can further impair the host antiviral immune response by disrupting the Janus kinase-STAT signaling pathway (8). Host proteins are crucial in counteracting viral invasion. For instance, a previous study suggested that the E3 ubiquitin ligase FANCL inhibits PPRV replication by promoting TBK1 phosphorylation (9).

The PPRV F protein is GC-rich and highly conserved. It mainly comprises transmembrane and cytoplasmic domains and regulates viral virulence (10). Additionally, the F protein forms fibrous projections on the viral envelope surface and induces cell fusion, which is a crucial requirement for successful viral infection; however, the activity of the F protein relies on its co-expression and interaction with the H protein (5, 11). After enzymatic cleavage, the F0 protein is processed into biologically active F1 and F2 fragments, and the study has found that the intracellular domain of the F0 protein can bind to the M protein, promoting viral budding (12, 13). And, purified F protein can cause lysis of chicken red blood cells, indicating that PPRV F protein also possesses hemolytic properties (14).

Human plasminogen activator urokinase (PLAU) is a serine protease belonging to Clan PA of S1 serine peptidases, which are mainly synthesized by cells such as macrophages, monocytes, and neutrophils in the lungs (15). Human PLAU regulates various biological activities to maintain normal activities in the body (16). Human PLAU functions as a serine protease and is primarily involved in proteolysis, extracellular matrix remodeling of proteins, growth factor activation, and plasminogen-to-plasminase conversion (17). Additionally, the interaction of PLAU with the N-terminus of the urokinase plasminogen activator receptor (uPAR/PLAUR) can trigger signal transduction inside and outside the cells (18). After binding to uPAR on the cell surface, human PLAU can activate tyrosine and serine/threonine kinases in the cell and the corresponding cell signal transduction pathway, thereby causing chemotaxis (19). Recent studies have found that human PLAU can activate the NF-κB signaling pathway and participate in the development of cholangiocarcinoma (20).

Interferons (IFNs) play a critical role in the early stages of the immune response. When pattern recognition receptors (PRRs) recognize pathogen-associated molecular patterns, an antiviral immune response is triggered, producing proinflammatory cytokines, chemokines, and IFN-I to combat viral invasion (21). Among the PRRs, retinoid acid-inducible gene I (RIG-I)-like receptors (RLRs) are the main receptors responsible for the production of IFNs, while PRRs are located in the cytoplasm and function as RNA sensors (22, 23). Although recent studies have reported that RLRs may also be located in the nucleus, they are located in the cytoplasm of most cells (24). Virus-induced signaling adapter (VISA) is an essential adapter protein in RLR signaling and can activate TBK1 and IκB kinase-ε, leading to the activation of IRF3 and IRF7. Subsequently, IRF3 and IRF7, together with the NF-κB, induce the transcription of IFN-I and other antiviral or immune-regulatory genes (25).

In our study, we found that goat PLAU can interact with PPRV F and that goat PLAU mainly inhibits viral replication by activating the VISA-triggered RLRs signaling pathway. Additionally, goat PLAU can antagonize the ability of F to degrade the VISA protein. These findings revealed a new mechanism by which host proteins regulate the innate immune response against PPRV replication.

## RESULTS

### PPRV inhibits the expression of goat PLAU

**Fig 1:**
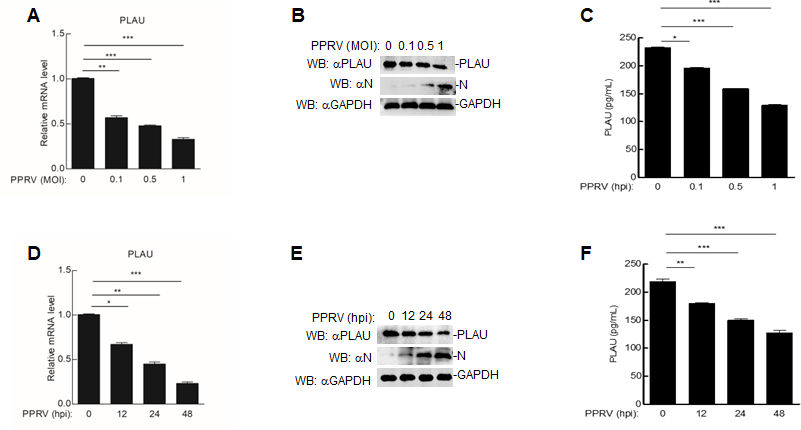
PPRV inhibits the expression of goat PLAU. (**A, B and C**) The effect of PPRV infection on mRNA, protein and concentration levels of goat PLAU in different doses. GAMs (2 × 10^6^) were infected with PPRV at different doses [multiplicity of infection (MOI) = 0, 0.1, 0.5, and 1.0] for 48 h. The levels of goat PLAU mRNA and protein were measured, and the PLAU concentration was measured using a Plasminogen Activator/Urokinase enzyme-linked immunosorbent assay ELISA KIT. (**D, E and F**) The effect of PPRV infection on goat PLAU mRNA, protein and concentration levels at different time points. GAMs (2 × 10^6^) were infected with PPRV (MOI = 1.0) for 0, 12, 24, and 48 h. The levels of goat PLAU mRNA and protein were measured, and the PLAU concentration was measured using a Plasminogen Activator/Urokinase ELISA KIT. Western blot analysis was performed using anti-PLAU antibodies. Data are means and SD (*n* = 3). *, *P* < 0.05; **, *P* < 0.01; ***, *P* < 0.001.

Currently, studies on regulating PPRV replication by host proteins are limited. We found that the mRNA and protein levels of goat PLAU decreased in a time- and dose-dependent manner in PPRV (Nigeria 75/1)-infected goat alveolar macrophages (GAMs) (Fig. 1A through F), prompting us to explore the function of goat PLAU in viral infection.

### PLAU interacts with F

**Fig 2:**
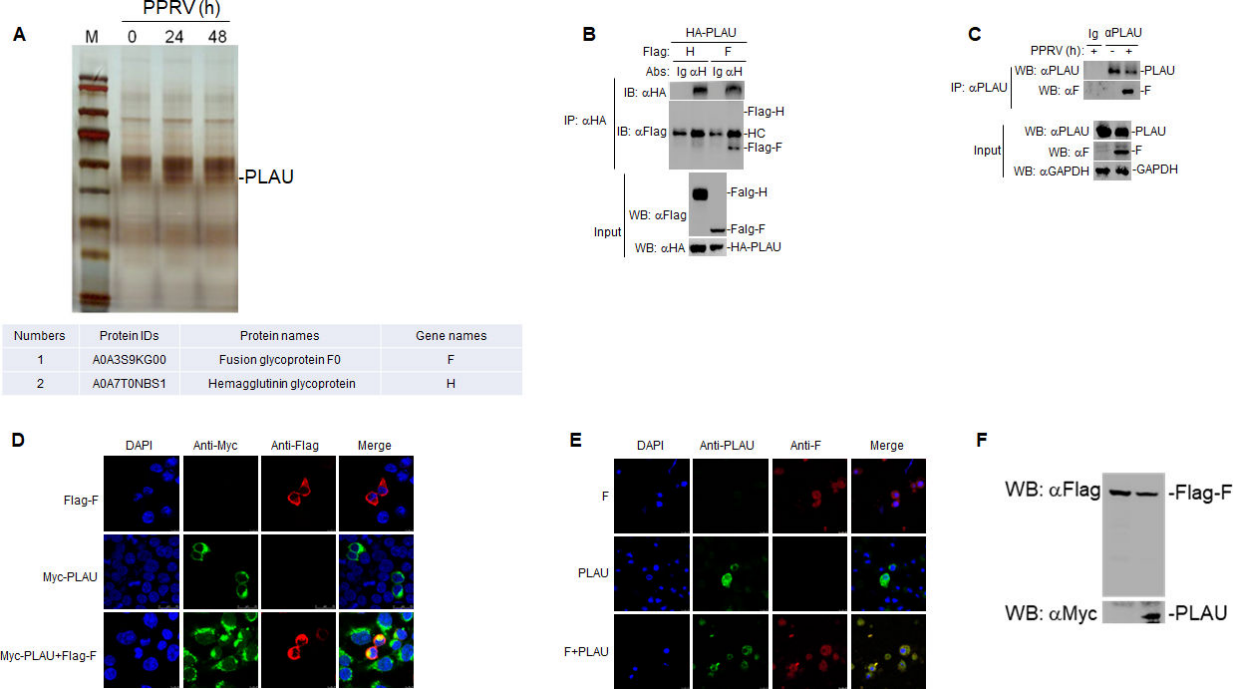
Goat PLAU interacts with F. (**A**) Goat PLAU interacts with PPRV H and F proteins. GAMs (2 × 10^7^) were infected with PPRV for 0, 24, and 48 h before the silver staining proteins after SDS electrophoresis in polyacrylamide gels and Co-IP were performed. (**B**) HA-PLAU interacts with Flag-F in HEK293T cells. HA-PLAU (5 µg) was co-transfected with Flag-F (5 µg) and Flag-H (5 µg) in HEK293T cells (2 × 10^6^), respectively. Co-IP with anti-HA antibody was performed, followed by western blot analysis. (**C**) Goat PLAU interacts with endogenous F in GAMs. GAMs (2 × 10^7^) were infected or not infected with PPRV (MOI = 5) for 48 h, and the cells were harvested. Co-IP with anti-PLAU antibody was performed, followed by western blot analysis. (**D**) Colocalization of HA-PLAU protein with Flag-F in HEK293T cells. HA-PLAU (0.5 µg) or Flag-F (0.5 µg) were individually transfected into HEK293T cells (4 × 10^5^) or co-transfected with HA-PLAU (0.5 µg) and Flag-F (0.5 µg) for 24 h. The cells were fixed overnight at 4°C and subjected to indirect immunofluorescence to detect HA-PLAU (green) and Flag-F (red) with mouse anti-HA and rabbit anti-Flag antibodies. The position of the nucleus is indicated by 4′,6-diamidino-2-phenylindole (DAPI) (blue) staining in the merged image. (**E**) Goat PLAU and F were colocalized in GAMs. After infection or non-infection with PPRV (MOI = 1.0) for 48 h in GAMs (2 × 10^6^). The cells were fixed overnight at 4°C and subjected to indirect immunofluorescence to detect PLAU (green) and F (red) with rabbit anti-PLAU and mouse anti-F antibodies. The position of the nucleus is indicated by DAPI (blue) staining in the merged image. (**F**) Goat PLAU has no function of cutting PPRV F protein. The HEK293T cells (4 × 10^5^) were transfected with Myc-PLAU (2 µg) and Flag-F (0.5 µg) for 24 h, followed by western blot analysis.

To further explore the interaction between goat PLAU and PPRV proteins, we conducted co-immunoprecipitation (Co-IP) followed by liquid chromatography–mass spectrometry (LC–MS). The results showed that goat PLAU interacted with PPRV H and F proteins (Fig. 2A). Subsequently, we co-transfected Flag-H and Flag-F with HA-PLAU into human embryonic kidney 293T (HEK293T) cells, respectively. Co-IP experiments revealed that HA-PLAU interacted with Flag-F but not with Flag-H (Fig. 2B). Goat PLAU and F exhibited endogenous interactions in PPRV-infected GAMs (Fig. 2C). Confocal microscopy further demonstrated that HA-PLAU and Flag-F exhibited exogenous colocalization in HEK293T cells (Fig. 2D) and that goat PLAU and F showed endogenous colocalization in PPRV-infected GAMs (Fig. 2E). Human PLAU has been shown to cleave the V3 ring in recombinant gp120 of human immunodeficiency virus type 1 IIIB and MN strains, as well as a synthetic cyclic peptide representing the V3 clay-B consistent sequence (26). To determine whether goat PLAU can cleave the F protein directly, we co-transfected Myc-PLAU and Flag-F expression plasmids into HEK293T cells. The results showed that goat PLAU could not directly cleave the PPRV F protein (Fig. 2F). These findings suggest that goat PLAU interacts with the PPRV F protein.

### PLAU inhibits the replication of PPRV

**Fig 3:**
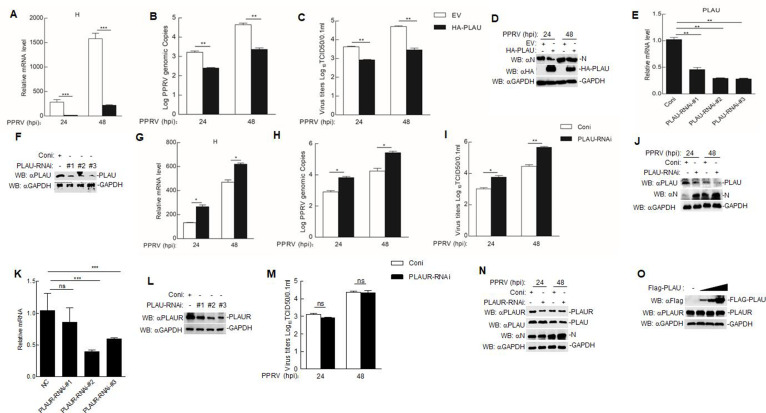
Goat PLAU inhibits the replication of PPRV. (**A to D**) Effect of goat PLAU overexpression on PPRV replication in Vero cells. In Vero cells (4 × 10^5^), HA-PLAU plasmid or EV (1 µg) was transfected for 24 h. Then, cells were followed by PPRV infection (MOI = 1.0) for 24 and 48 h. The levels of PPRV H gene mRNA (**A**), genomic copy numbers (**B**), virus titers (**C**), and N protein levels (**D**) were measured. (**E and F**) The effect of siRNA on goat PLAU expression level. GAMs (2 × 10^6^) were transfected separately with PLAU-RNAi-#1, PLAU-RNAi-#2, PLAU-RNAi-#3, and Control-RNAi (Coni) for 48 h. Then, the goat PLAU mRNA (**E**) and protein levels (**F**) were measured. (G–J) The effect of knocking down goat PLAU on PPRV replication in GAMs was investigated. The transfection mode of siRNA is shown in Fig. 3E. Then, cells were followed by PPRV infection (MOI = 1.0) for 24 and 48 h. The levels of PPRV H gene mRNA (**G**), genomic copy numbers (**H**), virus titers (**I**), and N protein levels (**J**) were measured. (**K and L**) The effect of siRNA on goat PLAUR expression level. The GAMs (2 × 10^6^) were transfected with PLAUR-RNAi-#1, PLAUR-RNAi-#2, PLAUR-RNAi-#3, and Coni for 48 h, respectively. Then, the goat PLAUR mRNA (**K**) and protein levels (**L**) were measured. (**M and N**) The effect of knocking down goat PLAUR on PPRV replication in GAMs was investigated. GAMs (2 × 10^6^) were transfected with PLAUR-RNAi and Coni for 48 h, and cells infected with PPRV (MOI = 1.0) for 24 or 48 h. PPRV virus titers (**M**) and N protein levels (**N**) were measured. (**O**) The effect of Flag-PLAU on PLAUR expression. Flag-PLAU (0, 0.5, 1, and 2 µg) was transfected into GAMs (2 × 10^6^) for 24 h, followed by western blot analysis. Data are means and SD (*n* = 3). *, *P* < 0.05; **, *P* < 0.01; ***, *P* < 0.001.

Given the decrease in goat PLAU expression following PPRV infection and the goat PLAU interacts with the PPRV F protein, we explored the effect of goat PLAU overexpression on PPRV replication. Vero cells were transfected with either HA-PLAU or an empty vector (EV) and then infected with PPRV. Our findings indicate that the mRNA levels of the PPRV H gene (Fig. 3A), genome copy numbers (Fig. 3B), virus titers (Fig. 3C), and PPRV N protein levels (Fig. 3D) were reduced in PPRV-infected Vero cells transfected with HA-PLAU.

To examine the role of endogenous goat PLAU in PPRV replication, we designed three pairs of specific siRNAs targeting goat PLAU in GAMs. Initially, we transfected GAMs with goat PLAU-targeted siRNAs and control-RNAi (Coni). The quantitative PCR (qPCR) and western blot results indicated that three pairs of goat PLAU-targeted siRNAs effectively reduced the expression of endogenous goat PLAU in GAMs (Fig. 3E and F). Subsequently, we transfected GAMs with either goat PLAU-RNAi or Coni and infected the cells with PPRV. We found that the mRNA levels of PPRV H (Fig. 3G), genome copy numbers (Fig. 3H), virus titers (Fig. 3I), and PPRV N protein levels (Fig. 3J) were increased in PPRV-infected goat PLAU-knockdown. The human PLAU–PLAUR interaction interferes with human immunodeficiency virus replication in monocyte-derived macrophages (27). To examine the role of endogenous goat PLAUR in PPRV replication, we designed three pairs of specific siRNAs targeting goat PLAUR in GAMs. Initially, we transfected GAMs with goat PLAUR-targeted siRNAs and Coni. The qPCR and western blot result showed that siRNAs targeting goat PLAUR effectively reduced the expression of goat endogenous PLAUR in GAMs (Fig. 3K and L). Subsequently, we transfected GAMs with goat PLAUR-RNAi or Coni and infected the cells with PPRV, respectively. We found that PPRV virus titers (Fig. 3M) and PPRV N protein levels (Fig. 3N) remained unchanged in PLAUR-knockdown GAMs and that PLAUR-knockdown did not affect PLAU expression in GAMs (Fig. 3N). We further overexpressed goat PLAU in GAMs and found that PLAU neither promoted nor inhibited PLAUR expression (Fig. 3O). These data suggest that goat PLAU expression, but not PLAUR, exerts an inhibitory effect on PPRV replication.

### Goat PLAU enhances the activation of RLR-mediated signaling

RLRs can collect specific intracellular junction proteins to initiate and activate signaling pathways such as NF-κB to control the transcription of genes encoding IFN-I and other inflammatory cytokines to inhibit virus replication (28). A previous study found that human PLAU could activate the NF-κB signaling pathway and participate in the development of bile duct cancer (20). We found high homology between human and goat PLAU using sequence alignment and investigated whether goat PLAU was involved in RLR-mediated signaling.

**Fig 4:**
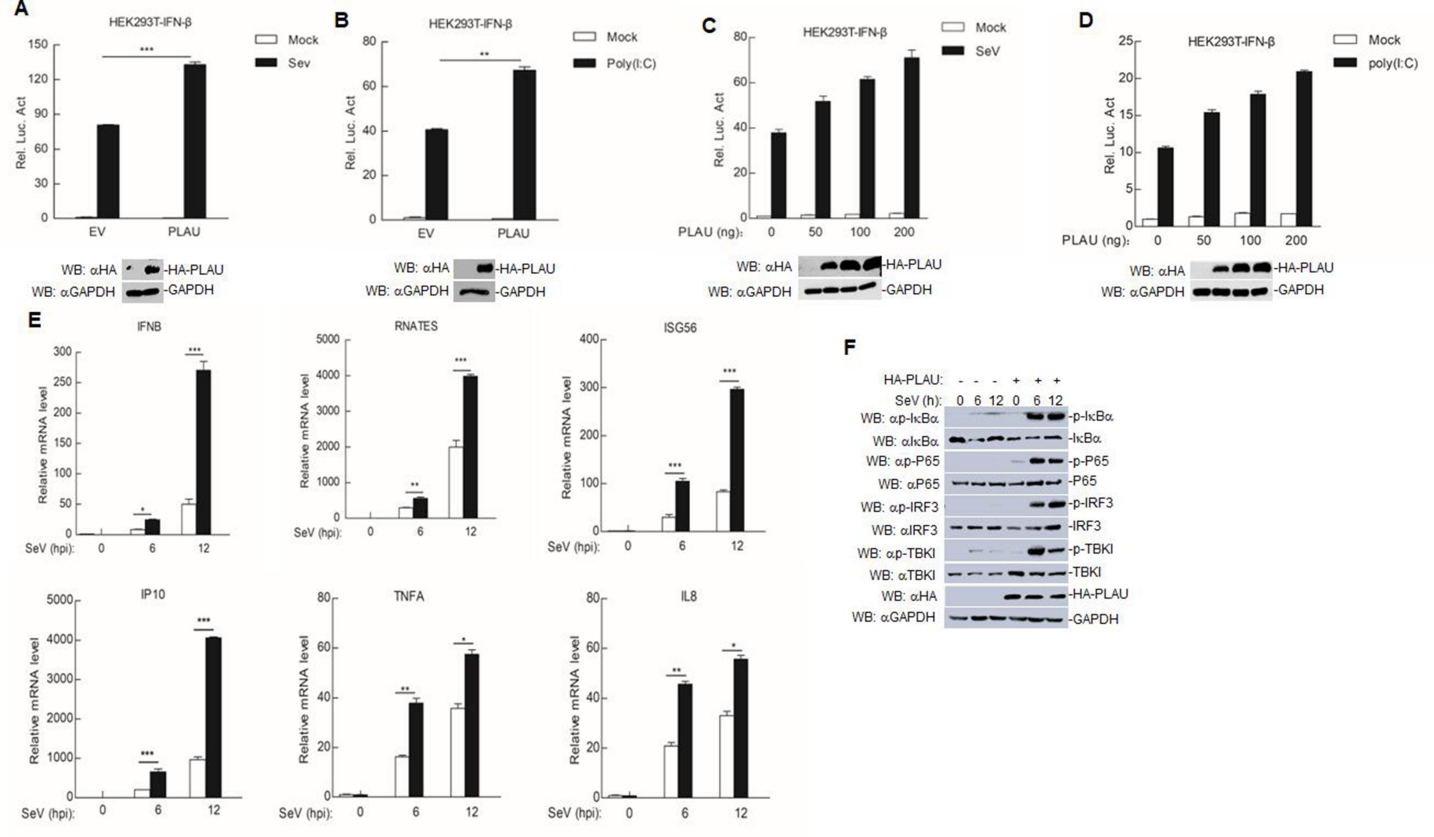
Goat PLAU enhances the activation of RLR-mediated signaling. (**A and B**) Effects of overexpression of goat PLAU on SeV or poly(I:C)-triggered IFN-β promoter activation. HEK293T cells (1 × 10^5^) were transfected with the IFN-β reporter (0.1 µg) and the indicated expression (0.1 µg) plasmids for 24 h. The cells were infected with SeV for 12 h or transfected with poly(I:C) for 18 h before luciferase assays were performed. (**C and D**) Dose-dependent effects of goat PLAU on SeV or poly(I:C)-triggered activation of IFN-β promoter. HEK293T cells (1 × 10^5^) were transfected with the IFN-β reporter (0.1 µg) and the indicated expression (0, 50, 100 and 200 µg) plasmids for 24 h. The cells were infected with SeV for 12 h or transfected with poly(I:C) for 18 h before luciferase assays were performed. (**E**) Goat PLAU promotes the induction of downstream antiviral genes triggered by SeV. HEK293T cells (4 × 10^5^) were transfected with HA-PLAU plasmid (1 µg) or EV plasmid (1 µg) for 24 h, followed by SeV infection. Subsequently, the transcription levels of downstream antiviral genes were detected using RT-PCR. (**F**) The effect of goat PLAU overexpression on the phosphorylation of IKBα, P65, IRF3, and TBK1 induced by SeV. The cells were treated similarly as in Fig. 4E, and western blot analysis was performed using specific antibodies. Data are means and SD (*n* = 3). *, *P* < 0.05; **, *P* < 0.01; ***, *P* < 0.001.

In reporter assays, we found that overexpressing goat PLAU in HEK293T cells enhanced the activation of the IFN-β promoter triggered by Sendai virus (SeV) and the RNA analog poly(I:C) (Fig. 4A and B). Subsequent experiments revealed that this activation increased dose-dependently in HEK293T cells (Fig. 4C and D). In a real-time PCR (RT-PCR) experiment, we observed that the transcription of several genes, including IFNB1, RANTES, ISG56, IP10, TNFA, and IL8, was elevated in goat PLAU-overexpressing SeV-infected HEK293T cells compared with that in control cells (Fig. 4E). Moreover, the overexpression of goat PLAU enhanced the phosphorylation of IκBα, P65, TBK1, and IRF3 (Fig. 4F), which are indicative of activated RLR-mediated signaling pathways. These results suggested that goat PLAU positively regulates RLR-mediated signaling.

### Goat PLAU interacts with VISA

**Fig 5:**
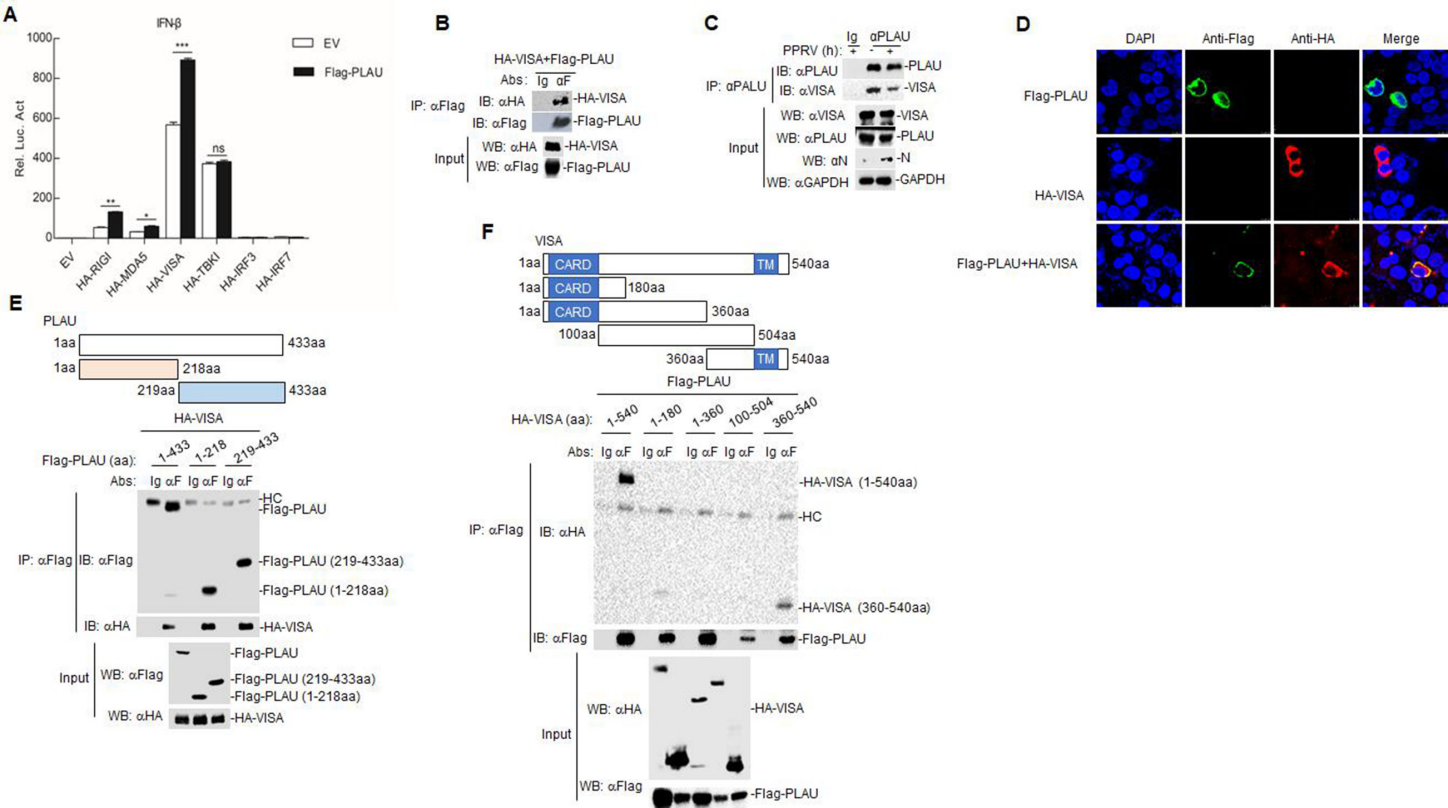
Goat PLAU interacts with VISA. (**A**) Goat PLAU targets upstream of TBKI. Effects of goat PLAU on IFN-β promoter activation by various signaling components. HEK293T cells (1 × 10^5^) were transfected with IFN-β promoter (0.1 µg) and expression plasmids for goat PLAU and the indicated proteins (0.1 µg each). Luciferase assays were performed 24 h after transfection. (**B**) The Flag-PLAU and HA-VISA interact with each other in HEK293T cells. Flag-PLAU (5 µg) and HA-VISA (5 µg) were co-transfected in HEK293T cells (2 × 10^6^). After 24 h of transfection, the cells were harvested. Co-IP was performed using anti-Flag antibody, followed by western blot analysis. (**C**) Endogenous associations between goat PLAU and VISA proteins in GAMs. GAMs (2 × 10^7^) were infected or not infected with PPRV (MOI = 5) for 48 h, and the cells were harvested. Co-IP was performed using anti-PLAU antibody, followed by western blot analysis. (**D**) Colocalization of Flag-PLAU protein with HA-VISA in HEK293T cells. Flag-PLAU (0.5 µg) or HA-VISA (0.5 µg) were individually transfected into HEK293T cells (4 × 10^5^) or co-transfected with Flag-PLAU (0.5 µg) and HA-VISA (0.5 µg) for 24 h. The cells were fixed overnight at 4°C and subjected to indirect immunofluorescence to detect Flag-PLAU (green) and HA-VISA (red) with mouse anti-Flag and rabbit anti-HA antibodies. The position of the nucleus is indicated by DAPI (blue) staining in the merged image. (**E**) Both the domains of Flag-PLAU (1–218 aa) and Flag-PLAU (219–433 aa) interact with VISA. Flag-PLAU (1–218 aa) (5 µg) and Flag-PLAU (219–433 aa) (5 µg) were co-transfected with HA-VISA (5 µg) in HEK293T, respectively. Co-IP was performed using anti-Flag antibody, followed by western blot analysis. (**F**) Flag-PLAU interacts with HA-VISA (360–540 aa). Flag-PLAU (5 µg) is used in HA-VISA (1–540), HA-VISA (1–180), HA-VISA (1–360), HA-VISA (100–504), and HA-VISA (360–540) (5 µg), respectively. Co-IP was performed using anti-Flag antibody, followed by western blot analysis. Data are means and SD (*n* = 3). *, *P* < 0.05; **, *P* < 0.01; ***, *P* < 0.001.

To determine the potential regulatory targets of goat PLAU, we investigated the effect of goat PLAU on the induction of the IFN-β promoter mediated by components of the RLR signaling pathway. Flag-PLAU, along with key signaling molecules (HA-RIG-I, HA-MDA5, HA-VISA, HA-TBK1, HA-IRF3, and HA-IRF7) on the RLR signaling pathway, and the IFN-β-Luc plasmid were co-transfected into HEK293T cells. Our results demonstrated that goat PLAU overexpression increased RIG-I-, MDA5-, and VISA-triggered the activation of the IFN-β promoter, but it had no effect on activation mediated by TBK1, IRF3, and IRF7 (Fig. 5A). Furthermore, we conducted a Co-IP assay using Flag-PLAU and HA-VISA co-transfected into HEK293T cells. The results revealed an interaction between the goat PLAU and VISA (Fig. 5B). Endogenous Co-IP experiments also confirmed the interaction between goat PLAU and VISA in PPRV-infected GAMs (Fig. 5C). In addition, we performed a colocalization analysis of goat PLAU and VISA by co-transfecting HEK293T cells with plasmids expressing Flag-PLAU and HA-VISA. Confocal microscopy showed that goat PLAU and VISA were colocalized (Fig. 5D).

To investigate the specific functional regions involved in the interaction between goat PLAU and VISA, we generated a series of functional domains for both proteins. The results showed that both the Flag-PLAU, Flag-PLAU [1–218 amino acid (aa)] and Flag-PLAU (219–433 aa) domains interacted with VISA (Fig. 5E). Additionally, goat PLAU interacted with full-length VISA as truncated VISA containing a C-terminal transmembrane domain (Fig. 4F). These findings support the hypothesis that goat PLAU interacts with VISA.

### Goat PLAU exerts its antiviral function through the domain PLAU (219–433 aa)

**Fig 6:**
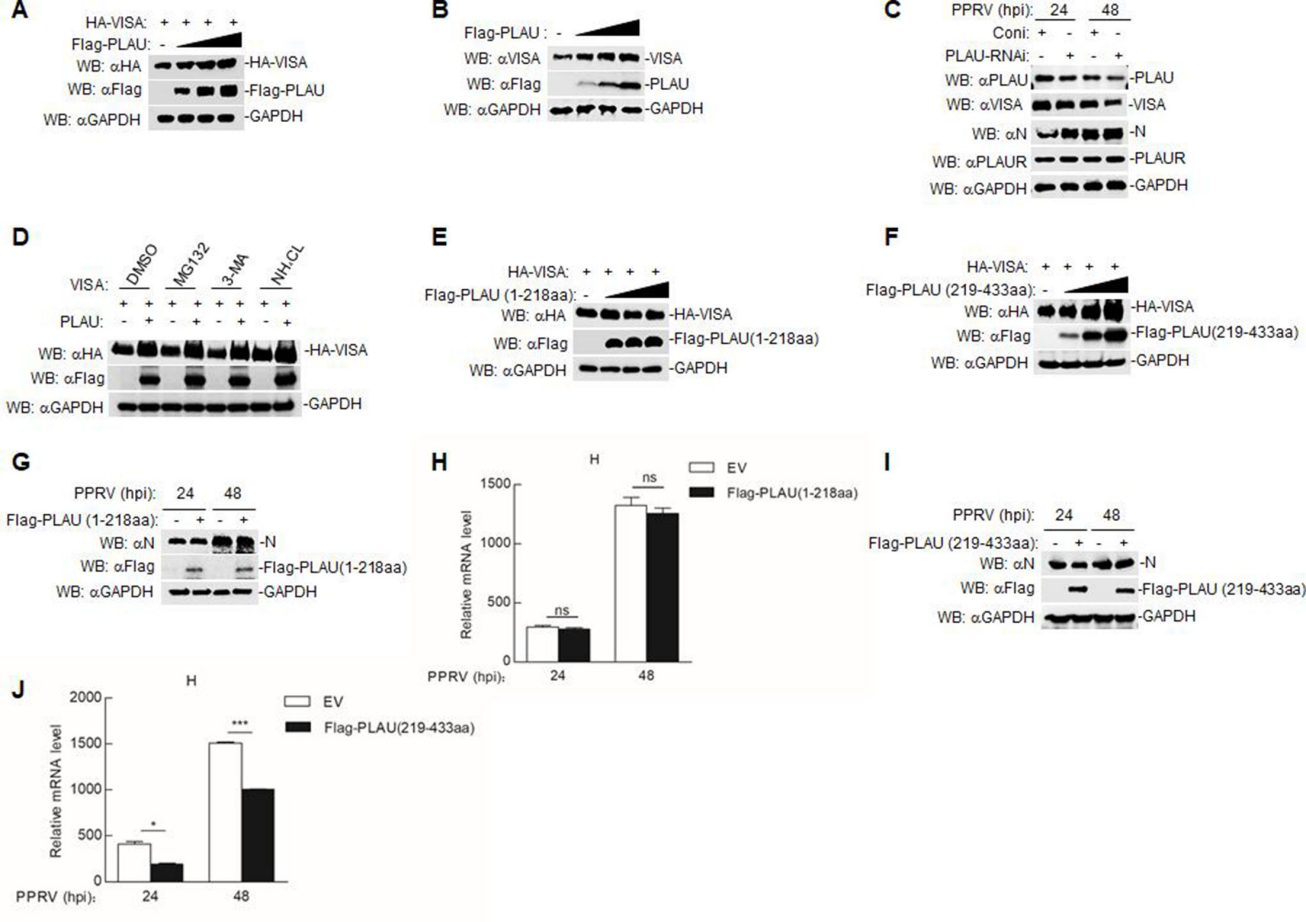
Goat PLAU exerts its antiviral function through the domain PLAU (219–433 aa). (**A**) The effect of Flag-PLAU on HA-VISA expression. The HEK293T cells (4 × 10^5^) were transfected with Flag-PLAU (0, 0.5, 1, and 2 µg) and HA-VISA (0.5 µg) for 24 h, followed by western blot analysis. (**B**) The effect of Flag-PLAU on endogenous VISA expression. Flag-PLAU (0, 0.5, 1, and 2 µg) was transfected into GAMs (2 × 10^6^) for 24 h, followed by western blot analysis. (**C**) The effect of goat PLAU-knockdown on endogenous VISA expression. SiRNA-treated cells were similar as in Fig. 3E. Then, cells were infected with PPRV (MOI = 1.0) for 24 or 48 h, followed by western blot analysis with specific antibodies. (**D**) Effects of inhibitors on the goat PLAU-mediated stability of VISA. The HEK293T cells (4 × 10^5^) were co-transfected with Flag-PLAU (1 µg) and HA-VISA (0.5 µg), and after transfection for 18 h, the cells were treated with the specific inhibitor for 6 h, and then western blot analysis was performed. (**E and F**) The effect of goat PLAU domains on VISA expression. Flag-PLAU (1–218 aa) or Flag-PLAU (219–433 aa) (0, 0.5, 1, and 1.5 µg) were co-transfected with HA-VISA (0.5 µg) in HEK293T cells (4 × 10^5^) for 24 h, followed by western blot analysis. (**G and H**) The effect of Flag-PLAU (1–218aa) on PPRV replication. Vero cells (4 × 10^5^) were transfected with Flag-PLAU (1–218 aa) (1 µg) for 24 h, followed by PPRV (MOI = 1.0) infection for 24 and 48 h. The levels of PPRV N protein and H gene mRNA were then measured. (**I and J**) The effect of Flag-PLAU (219–433aa) (1 µg) on PPRV replication. It is processed in the same way as Fig. 6G and H.

Having established an interaction between goat PLAU and VISA, we explored how goat PLAU regulates VISA. Co-transfecting Flag-PLAU and HA-VISA into HEK293T cells revealed that Flag-PLAU augmented HA-VISA expression in a dose-dependent manner (Fig. 6A). In addition, we found that the expression of endogenous VISA increased in goat PLAU-overexpression GAMs (Fig. 6B). Upon PPRV infection of goat PLAU-knockdown GAMs, we observed a decrease in VISA expression and a surge in PPRV replication; however, PLAUR expression remained unchanged (Fig. 6C). To investigate the mechanisms by which goat PLAU affects the stability of VISA, we treated the cells with various inhibitors of protein degradation pathways. The lysosome inhibitor, ammonium chloride (NH_4_Cl), but not the proteasome inhibitor (MG132) or autophagy pathway inhibitor (3-MA), inhibited the increase in VISA (Fig. 6D). Building on our previous finding that both the Flag-PLAU (1–218 aa) and Flag-PLAU (219–433 aa) domains interact with VISA (Fig. 4E), we separately overexpressed these domains in HEK293T cells. Only the Flag-PLAU (219–433 aa) domain influenced VISA expression (Fig. 6E and F). Overexpression of this domain in Vero cells uniquely suppressed PPRV N protein expression and H mRNA levels (Fig. 6G through J). These findings highlight the significance of the PLAU (219–433 aa) domain in its antiviral role.

### Interaction among goat PLAU, VISA, and PPRV F Protein

**Fig 7:**
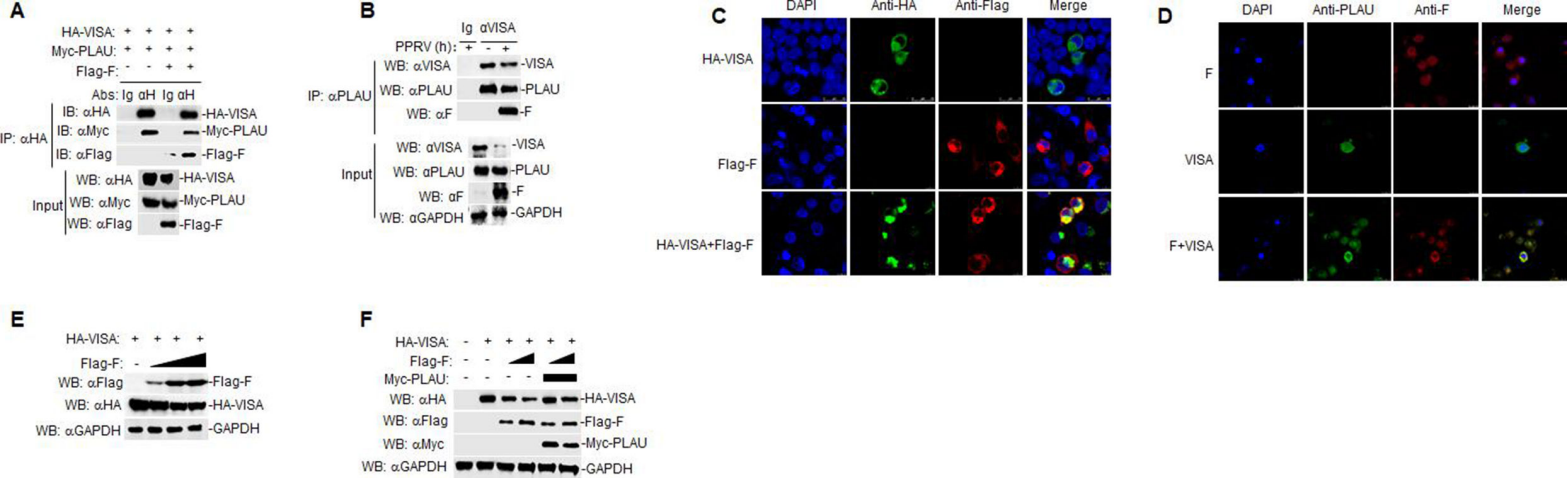
Goat PLAU, VISA, and PPRV F proteins interact with each other. (**A**) Myc-PLAU, HA-VISA, and Flag-F proteins interact with each other. The HEK293T cells (2 × 10^6^) were transfected with Myc-PLAU (5 µg), HA-VISA (5 µg), and Flag-F (5 µg). After 24 h of transfection, the cells were harvested. Co-IP was performed using anti-HA antibody, followed by western blot analysis. (**B**) VISA can interact with goat PLAU and F endogenous. GAMs (2 × 10^7^) were infected or not infected with PPRV (MOI = 5) for 48 h, and the cells were harvested. Co-IP used anti-VISA, followed by western blot analysis. (**C**) Colocalization of HA-VISA with Flag-F in HEK293T cells. HA-VISA (0.5 µg) or Flag-F (0.5 µg) were individually transfected into HEK293T cells (4 × 10^5^) or co-transfected with HA-PLAU (0.5 µg) and Flag-F (0.5 µg) for 24 h. The cells were fixed overnight at 4°C and subjected to indirect immunofluorescence to detect HA-VISA (green) and Flag-F (red) with mouse anti-HA and rabbit anti-Flag antibodies. The position of the nucleus is indicated by DAPI (blue) staining in the merged image. (**D**) VISA and F were colocalized in GAMs. After infection or non-infection with PPRV (MOI = 1.0) for 48 h in GAMs (2 × 10^6^). The cells were fixed overnight at 4°C and subjected to indirect immunofluorescence to detect VISA (green) and F (red) with rabbit anti-VISA and mouse anti-F antibodies. The position of the nucleus is indicated by DAPI (blue) staining in the merged image. (**E**) The effect of Flag-F on HA-VISA expression. Flag-F or HA-VISA (0, 0.5, 1, and 1.5 µg) were co-transfected with HA-VISA (0.5 µg) in HEK293T cells (4 × 10^5^) for 24 h, followed by western blot analysis. (**F**) Goat PLAU can restore the degradation of VISA protein by F. Immunoblotting analysis of HEK293T cells (4 × 10^5^) co-transfected with plasmids expressing F (0.5 or 1 µg; wedges), PLAU protein (2 µg; black rectangular box), and VISA (0.5 µg) (+) or empty vectors (−).

To confirm the interaction among F, goat PLAU, and VISA, we co-transfected Myc-PLAU, HA-VISA, and Flag-F plasmids into HEK293T cells. These results indicate that VISA interacts with both goat PLAU and F (Fig. 7A). Endogenous Co-IP showed that VISA also interacted with goat PLAU and F in PPRV-infected GAMs (Fig. 7B). Confocal experiments showed exogenous colocalization of HA-VISA and Flag-F in HEK293T cells (Fig. 7C) and endogenous colocalization of VISA and F in PPRV-infected GAMs (Fig. 7D). In the following experiments, we also demonstrated that F could degrade VISA (Fig. 7E). Because of the interaction among F, VISA, and goat PLAU, we conducted co-transfection experiments in HEK293T cells and found that goat PLAU could restore the degradation of VISA caused by the PPRV F protein (Fig. 6F).

### The inhibitory effect of goat PLAU on PPRV replication is weakened in VISA-knockdown GAMs

**Fig 8:**
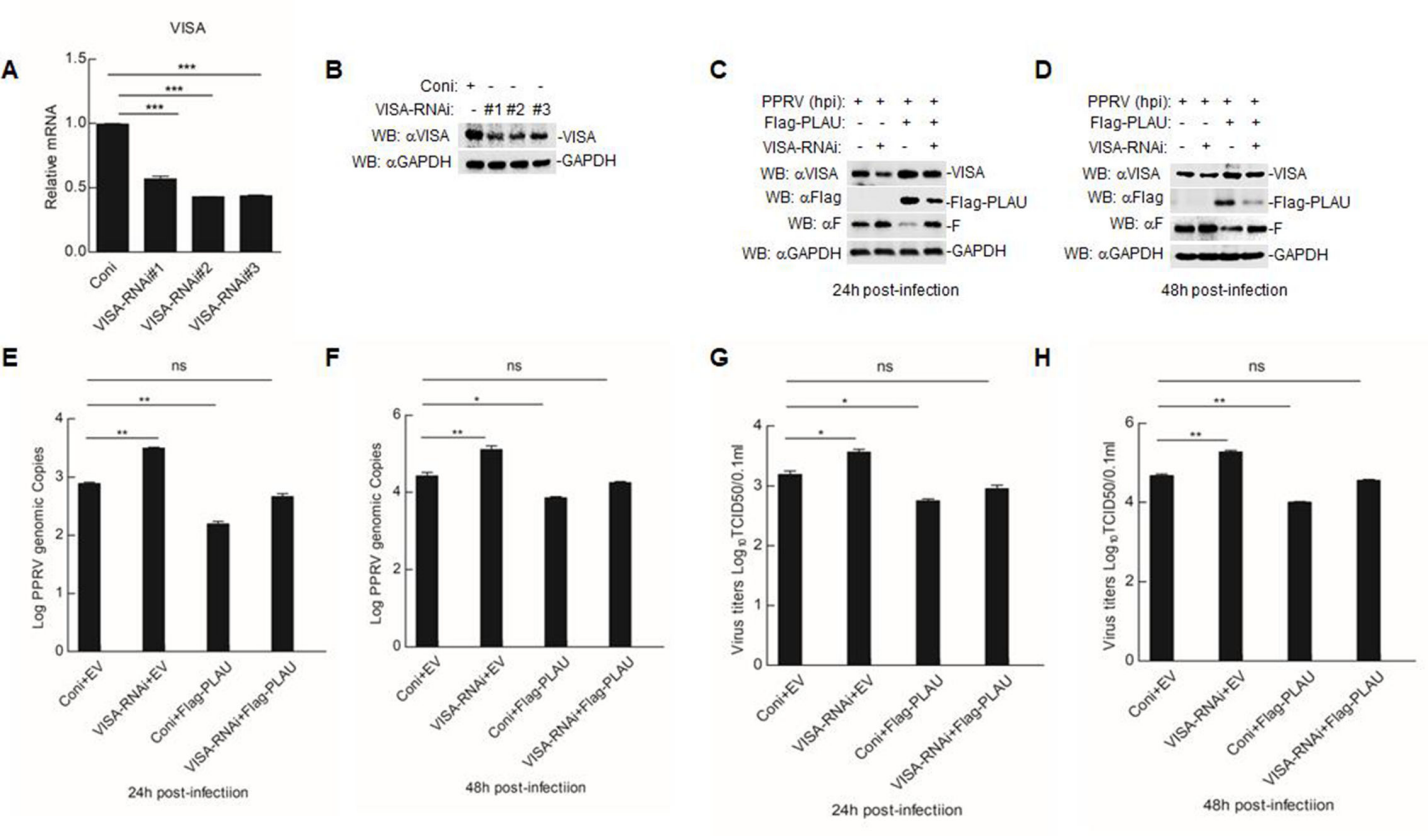
The inhibitory effect of goat PLAU on PPRV replication is weakened in VISA-knockdown GAMs. (**A and B**) The effect of siRNA on goat VISA expression level. The GAMs (2 × 10^6^) were separately transfected with VISA-RNAi-#1, VISA-RNAi-#2, VISA-RNAi-#3, and Coni for 48 h. Then, the VISA mRNA (**A**) and protein levels (**B**) were measured. (**C to H**) The inhibitory effect of goat PLAU on PPRV replication is weakened in VISA-knockdown GAMs. VISA-RNAi was transfected in GAMs (2 × 10^6^) for 48 h, followed by Flag-PLAU transfection for 24 h. Then, the cells infected PPRV (MOI = 1.0) for 24 and 48 h, respectively. The levels of PPRV N protein levels (**C and D**), genomic copy numbers (**E and F**), and virus titers (**G and H**) were measured. Data are means and SD (*n* = 3). *, *P* < 0.05; **, *P* < 0.01; ***, *P* < 0.001.

To elucidate the role of endogenous VISA in PPRV infection, we constructed three pairs of siRNAs targeting goat VISA to inhibit its endogenous expression in GAMs. These siRNAs markedly reduced endogenous VISA expression (Fig. 8A and B). To determine the effect of goat PLAU on PPRV replication in VISA-knockdown GAMs, VISA-knockdown GAMs were transfected with Flag-PLAU, followed by PPRV infection. The results showed that the ability of goat PLAU to inhibit PPRV replication was impaired in the VISA-knockdown GAMs. These results suggest that VISA plays a role in promoting the antiviral effects of PLAU in goat GAMs (Fig. 8C through H).

## DISCUSSION

Human PLAU was first isolated from urine and is involved in plasminogen activation by serine protein hydrolase. It has various functions and can affect the biological behavior of the body in various manners (29, 30). The expression of human PLAU gradually increases with age, which is used as a biomarker of age, and is also involved in inflammatory responses (31–33). In addition, human PLAU can activate receptor kinases and signaling pathways on the cell surface, such as the epidermal growth factor receptor (34) and protein kinase B (35).

In this study, we found that the mRNA and protein levels of goat PLAU decreased with an increase in the PPRV infection time or dose (Fig. 1). This finding led us to speculate that goat PLAU plays a regulatory role in PPRV infection. We confirmed the interaction between goat PLAU and F by LC-MS, Co-IP, and confocal microscopy (Fig. 2). We further demonstrated that goat PLAU inhibits PPRV replication, whereas PLAUR does not (Fig. 3).

An essential component of the body’s defense system is the IFN system, which mediates various biological effects. They can inhibit viral growth non-specifically by inducing an “antiviral state” in cells (36). In the IFN system, this function is mainly mediated by IFN-I. Human PLAU has been shown to activate inflammatory responses in the NF-κB pathway in previous studies, while RIG-I or MDA5 signaling pathways have been shown to induce NF-κB or IRF3-mediated IFN-I production after viral stimulation, and IFN-I activates the JAK-STAT signaling pathway (20, 37, 38). Therefore, we suspected that goat PLAU influenced PPRV replication by promoting IFN-I. Next, we explored whether goat PLAU inhibited viral replication by promoting IFN-I production. As shown in Fig. 4, overexpression of goat PLAU significantly enhanced SeV and poly(I:C)-induced IFN-β promoter activation in a dose-dependent manner. It also promoted the phosphorylation of downstream genes like IκBα, P65, TBK1, and IRF3, indicating the activation of the RLR pathway.

RLRs, which are composed of three members (RIG-I, MDA5, and LGP2), are precise sensors of viral invasion, it can distinguish between autologous and non-autologous cells to avoid uncontrolled automatic activation of the congenital immune system without viral infection (39). Upon activation, RLRs move to the mitochondria and associated membranes, activating VISA, which initiates downstream IFNs- and interferon-stimulating gene (ISGs) induction (40). Our results indicate that goat PLAU augments IFN-β promoter activation mediated by RIG-I, MDA5, and VISA but not TBK1, IRF3, and IRF7, suggesting goat PLAU targets proteins upstream of TBK1 (Fig. 5A). We further confirmed that goat PLAU interacts with VISA (Fig. 5B through F).

VISA, a downstream adaptor protein of RLRs, has been a hot research topic regarding signal transduction and regulation of innate immunity. Although we have demonstrated an interaction between goat PLAU and VISA, the mechanism by which goat PLAU affects VISA and subsequently modulates innate immunity requires further research. Next, goat PLAU was overexpressed in HEK293T cells and GAMs. The results showed that goat PLAU increased VISA protein expression dose-dependently (Fig. 6A and B). The expression of VISA was reduced in goat PLAU-knockdown GAMs (Fig. 6C). Inhibitor experiments further confirmed that ammonium chloride treatment could restore goat PLAU-mediated promotion of VISA, indicating that goat PLAU maintains the stability of VISA through the lysosomal pathway (Fig. 6D), thus affecting the regulation of natural immunity by VISA. As shown in Fig. 6E through J, we further confirmed that goat PLAU exerted antiviral effects through the functional domain of the PLAU (219–433 aa).

Viruses employ various strategies to evade the host immune system, promoting viral pathogenicity and immune evasion (41, 42). During this process, viral proteins play a crucial role in inhibiting IFN-I production by creating a favorable environment for viral replication. For example, PPRV inhibits IFN-I response and promotes immune evasion by targeting IRAKI (43). Foot-and-mouth disease virus (FMDV) non-structural protein 3A interacts with G3BP1, upregulating the expression of the autophagy-related protein LRRC25 and suppressing the expression of G3BP1 and the G3BP1-mediated RLH signaling pathway (44). Additionally, the structural protein VP3 of the FMDV interacts with Rab7b to promote FMDV replication (45). Furthermore, VP3 is a negative regulator of the virus-induced IFN-β signaling pathway (46). However, the molecular mechanism by which PPRV proteins affect replication capacity is not fully understood. Among all PPRV proteins, the F protein is a key factor in the regulation of viral virulence; therefore, a preliminary study of PPRV F will contribute to an in-depth understanding of the replication mechanism of PPRV. Herein, we found that F can interact with goat PLAU/VISA and inhibit VISA expression, creating a favorable environment for the replication of PPRV. However, goat PLAU restored VISA degradation caused by F (Fig. 7). Simultaneously, we found that PPRV replication increased, and the inhibitory effect of goat PLAU on PPRV replication was weakened in VISA-knockdown GAMs (Fig. 8). Goat PLAU inhibited viral replication by targeting VISA. Based on the above results, we confirmed that the PPRV F protein can evade host immunity by inhibiting the expression of VISA. In contrast, goat PLAU activates the RLRs signaling pathway targeting VISA, restores the function of the F protein to degrade VISA, and inhibits the growth of PPRV.

This study sheds light on the molecular mechanisms underlying goat PLAU-mediated innate immune response during PPRV replication. Specifically, goat PLAU inhibits PPRV by targeting the VISA-mediated RLRs signaling pathway and counteracts the ability of the PPRV F protein to degrade VISA. These findings provide a theoretical basis for further studies on the interactions between PPRV and host proteins and offer insights for developing new PPRV vaccines.

## MATERIALS AND METHODS

### Cell culture and virus infection

GAMs were isolated from a 6-weeks-old goat. GAMs were cultured in RPMI 1640 medium containing 10% fetal bovine serum (FBS) (Gibco) and 1% penicillin–streptomycin. HEK293T cells were cultured in Dulbecco’s modified Eagle’s medium (Invitrogen, Waltham, MA, USA) supplemented with 1% penicillin–streptomycin–gentamicin solution and 10% FBS. All cells were cultured at 37°C under a 5% CO_2_ atmosphere saturated with water. The attenuated PPRV strain Nigeria 75/1 was obtained in our laboratory using Vero cells. The viral load was confirmed as CPE and quantified by determining the 50% tissue culture infective dose (TCID_50_). The multiplicity of infection (MOI) was confirmed based on the viral titers of the respective cell lines. For viral infection, GAMs were seeded into 6-well cell culture plates at a density of 2 × 10^6^ cells/mL and inoculated with or without PPRV (Nigeria 75/1). After adsorption for 2 h, the infected cells were maintained in RPMI 1640 medium (containing 2% FBS).

### Plasmid construction

The Flag-tagged expression plasmids of H and F used in the present study have been described previously(47), and the mammalian expression plasmids for Flag-PLAU and Myc-PLAU were constructed using standard molecular biology techniques. Mammalian expression plasmids for Flag-PLAU (1–218 aa) and Flag-PLAU (219–433 aa) were constructed using the Flag-PLAU plasmid as a template. The primers are listed in Table 1.

**TABLE 1:** Primers used in this study

Primers	Sequences (5′–3′)
Human IP10	F: GGTGAGAAGAGATGTCTGAATCC R: GTCCATCCTTGGAAGCACTGCA
Human IL8	F: GAGAGTGATTGAGAGTGGACCAC R: CACAACCCTCTGCACCCAGTTT
Human TNFA	F: GCCGCATCGCCGTCTCCTAC R: CCTCAGCCCCCTCTGGGGTC
Human ISG56	F: TCATCAGGTCAAGGATAGTC R: CCACACTGTATTTGGTGTCTAGG
Human IFNB	F: TTGTTGAGAACCTCCTGGCT R: TGACTATGGTCCAGGCACAG
Human RANTES	F: GGCAGCCCTCGCTGTCATCC R: GCAGCAGGGTGTGGTGTCCG
Human GAPDH	F: GAGTCAACGGATTTGGTCGT R: GACAAGCTTCCCGTTCTCAG
Goat PLAU	F: GCGGTGGCAGTCTCATCAGTC R: GCAAGGCAATATCGTTGTGGTGAG
Goat GAPDH	F: CACTGCCACCCAGAAGACT R: CAGATCCACAACGGACACG
Flag-PLAU	F: TTACAAGGATGACGATGACAAGCTTATGAGGGTCCTGTTGGCATGCCT R: GCCACCCGGGATCCTCTAGAGTCGACTCACAGGCCAAGGTCAATCTCT
Flag-PLAU (1–218 aa)	F: TTACAAGGATGACGATGACAAGCTTATGAGGGTCCTGTTGGCATGCCT R: CCACCCGGGATCCTCTAGAGTCGACTCAGGGACTGATGAGACTGCCAC
Flag-PLAU (219–433 aa)	F: TACAAGGATGACGATGACAAGCTTATGTGCTGGGTGGTCAGCGCCAC R: GCCACCCGGGATCCTCTAGAGTCGACTCACAGGCCAAGGTCAATCTCT
Myc-PLAU	F: AGCTGGCTAGTTAAGCTTATGAGGGTCCTGTTGGCAT R: GAGAGATTGACCTTGGCCTGTCTAGAGGGCCCTTCGAA
Goat VISA	R: GCATCAGGAGCAGGACACAGAAC F: TGGAAGGAGACAGATGGAGACACAG
PPRV H	F: CTTGGAGTCCTGCTGGTAATGTTC R: GCCGATTCTGGATCTCTGAAGTTC
Green monkey GAPDH	F: ACCACAGTCCACGCCATCAC R: TGACCTTGCCCACAGCCTTG

### Antibodies and reagents

Monoclonal mouse anti-HA (H3663), anti-Myc (SAB1305535), and anti-Flag (F3040) antibodies were purchased from Sigma-Aldrich (USA). Polyclonal rabbit anti-IκBα, p-IκBα, P65, p-P65, IRF3, p-IRF3, TBK1, and p-TBK1 were purchased from Cell Signaling Technology (USA). GAPDH, anti-mouse IgG, and anti-rabbit IgG were obtained from Thermo Scientific. Antibodies against PLAU, PLAUR and VISA were purchased from Proteintech. CoraLite594-conjugated goat anti-mouse IgG (H + L) and fluorescein isothiocyanate (FITC)-conjugated goat anti-rabbit IgG (H + L) antibodies were purchased from CST. 3-Methyladenine (3-MA) (Catalogue #189490) and MG132 (M8699) were purchased from Sigma-Aldrich. The Plasminogen Activator/Urokinase enzyme-linked immunosorbent assay (ELISA) KIT was purchased from Solarbio.

### Transfection and reporter gene assays

HEK293T cells (1 × 10^5^) were seeded in 48-well plates and transfected using standard calcium phosphate precipitation the following day. In the same experiment, an empty control plasmid was added to ensure that each transfection received the same amount of total DNA. To normalize transfection efficiency, 10 ng of the pRL-TK Renilla luciferase reporter plasmid was added to each transfection. Luciferase assays were performed with a dual-specific luciferase assay kit (Promega). Firefly luciferase activity was measured and normalized to the Renilla luciferase activity.

### Quantitative real-time PCR

Total RNA was isolated using TRIzol reagent (Sigma, USA) and quantified using a NanoDrop ND-2000C spectrophotometer (Wilmington, DE, USA). cDNA was synthesized from 1 µg of RNA using a GoScript reverse transcription system (Promega, Madison, WI, USA). The cDNA of all targeted gene transcripts was quantified using the SYBR green method. A 25 µL reaction mixture included 12.5 µL of 2× SYBR mix, 0.5 µL (10 µM) forward primer, 0.5 µL (10 µM) reverse primer, 10.5 µL H_2_O, and 1 µL of cDNA product from the first round. The PCR cycling conditions were 95°C for 10 min, followed by 40 cycles of 95°C for 15 s and 60°C for 1 min. GAPDH was used as a housekeeping gene. The relative expression of targeted genes was calculated using the 2^−ΔΔCT^ method and is presented as fold change. The primers for RT-PCR are listed in Table 1.

### siRNA knockdown

siRNAs corresponding to goat PLAU, PLAUR and VISA target sequences were purchased from Sangon Biotech (China). GAMs were transfected with Coni and PLAU-RNAi, Coni and PLAUR-RNAi or Coni and VISA-RNAi using the jetPRIME reagent for 48 h. The siRNA sequences are listed in Table 2.

**TABLE 2:** SiRNAs used in this study

siRNA	Sequences (5'–3')
Goat PLAU-RNAi#1	F: GCAGCAAUGAAGUUCAUAATT R: UUAUGAACUUCAUUGCUGCTT
Goat PLAU-RNAi#2	F: CCACACACUGCUUCAUUAATT R: UUAAUGAAGCAGUGUGUGGTT
Goat PLAU-RNAi#3	F: CCGCAACAAUCACAGCAAUTT R: AUUGCUGUGAUUGUUGCGGTT
Goat PLAUR-RNAi#1	F: GGGCUGGAAAUCAGAUCAUTT R: AUGAUCUGAUUUCCAGCCCTT
Goat PLAUR-RNAi#2	F: GCAACACCACCAAAUGCAATT R: UUGCAUUUGGUGGUGUUGCTT
Goat PLAUR-RNAi#3	F: GCUCUUGACUGCCAGACUUTT R: AAGUCUGGCAGUCAAGAGCTT
Goat VISA-RNAi#1	F: CCGCAACAAUCACAGCAAUTT R: AUUGCUGUGAUUGUUGCGGTT
Goat VISA-RNAi#2	F: GGACCUCUUCGACAGUCUUTT R: AAGACUGUCGAAGAGGUCCTT
Goat VISA-RNAi#3	F: CCCAUCAACUCAGUGCGUUTT R: AACGCACUGAGUUGAUGGGTT

### Co-IP and western blotting

For transient-transfection and Co-IP experiments, HEK293T cells (2 × 10^6^) were transfected with the respective plasmids for 24 h. Then, they were lysed in 1 mL of lysis buffer [20 mM Tris (pH 7.5) 150 mM NaCl, 1% Triton X-100, 1 mM EDTA, 10 µg/mL aprotinin, 10 µg/mL leupeptin, and 1 mM phenylmethylsulfonyl fluoride]. For each immunoprecipitation, a 0.4 mL aliquot of lysate was incubated with 0.2 µg of the indicated antibodies or control IgG and 25 µL of 1:1 slurry of Gammabind G plus Sepharose (Amersham Biosciences) for 2 h. Sepharose beads were washed thrice with 1 mL of lysis buffer containing 500 mM NaCl. The precipitates were analyzed by western blotting as previously described (45). For endogenous Co-IP experiments, GAMs (2 × 10^7^) were infected with PPRV for the indicated periods. Co-IP and immunoblotting were performed as previously described.

### Confocal microscopy

HEK293T cells (4 × 10^5^) were seeded in 35 mm confocal glass-bottom dishes for confocal imaging. GAMs (2 × 10^6^) were infected with PPRV (MOI = 1.0) for 48 h. The cells were fixed overnight at 4% paraformaldehyde at 4°C. After washing three times with phosphate-buffered saline (PBS), the cells were permeabilized with PBS containing 0.1% Triton X-100 for 10 min at room temperature, washed three times with PBS, and blocked with PBS containing 5% bovine serum albumin at 37°C for 30 min. The cells were incubated with a specific antibody (1:200) at 4°C overnight. After incubation with the secondary antibody, the cells were stained with 4′,6-diamidino-2-phenylindole (DAPI) for 5 min. Finally, the stained cells were visualized under a confocal laser microscope (Leica, Germany).

### Statistical analysis

Data graphs were created using GraphPad Prism 5.0. Data were analyzed using SPSS 16.0 using one-way analysis of variance. A *P* value of <0.05 was considered statistically significant.
